# Genetic Relatedness of Low Solitary Nests of *Apis dorsata* from Marang, Terengganu, Malaysia

**DOI:** 10.1371/journal.pone.0041020

**Published:** 2012-07-20

**Authors:** Najmeh Sahebzadeh, Makhdzir Mardan, Abdul Manaf Ali, Soon Guan Tan, Nur Azura Adam, Wei Hong Lau

**Affiliations:** 1 Department of Plant Protection, Faculty of Agriculture. Universiti Putra Malaysia, Serdang, Selangor, Malaysia; 2 Department of Agriculture Technology, Faculty of Agriculture, Universiti Putra Malaysia, Serdang, Selangor, Malaysia; 3 Department of Biotechnology, Faculty of Agriculture and Biotechnology, Universiti Sultan Zainal Abidin, Kuala Terengganu, Terengganu, Malaysia; 4 Department of Cell and Molecular Biology, Faculty of Biotechnology and Biomolecular Sciences, Universiti Putra Malaysia, Serdang, Selangor, Malaysia; UCLA, United States of America

## Abstract

Knowledge on the population of genetic structure and ecological behaviour of *Apis dorsata* from Peninsular Malaysia is needed for effective management and conservation of this species since unsustainable whole solitary low nest cutting for product harvesting is the current common practice here. The analysis of 15 single locus DNA microsatellite markers on samples from 20 solitary nests of *A. dorsata* showed that while these markers were polymorphic, high intracolonial relatedness existed. Furthermore, in general, slightly negative values of intercolony relatedness (R) among the nests of *A. dorsata* were found. However, positive values of mean intercolony relatedness were observed between 54 pairs of nests out of 190 possible combinations. The R values among nest pairs 3–4 and 3–5 was higher than 0.50 showing that their queens were half siblings, whereas nest pair 19–20 showed relatedness of 0.95 indicating that the same queen was sampled. The results that we obtained could not conclusively support the hypothesis of this study that the honey hunters in Marang district of Malaysia repeatedly harvest the same nest located at a different site and at a different time during the same honey harvesting season. However, our finding of an appreciable level of intercolonial relatedness between several pairs of nests in this pioneer study indicated that a comprehensive study with a larger sample size of solitary nests found throughout the region would be necessary to provide concrete proof for this novel idea.

## Introduction

The Indo-Malaya region is known as the centre of honeybee diversity, whereby seven out of the nine honeybee species of the world are sympatric and endemic to this region [1]. Among the *Apis* species, the nests of *A. dorsata* are known as the sources of wild honey in Malaysia [2]. Many colonies of the single-comb, open-nesting and sedentary of *A*. *dorsata* are found to nest either singly or low to the ground, or high in aggregates on tree limbs of tall bee trees along the coastal, submerged *Melaleuca* forest in Marang, Terengganu, Malaysia [3]. Of the two nest types, it is the low solitary nests that are usually harvested in Peninsular Malaysia and thus is the subject of this study.

**Figure 1:**
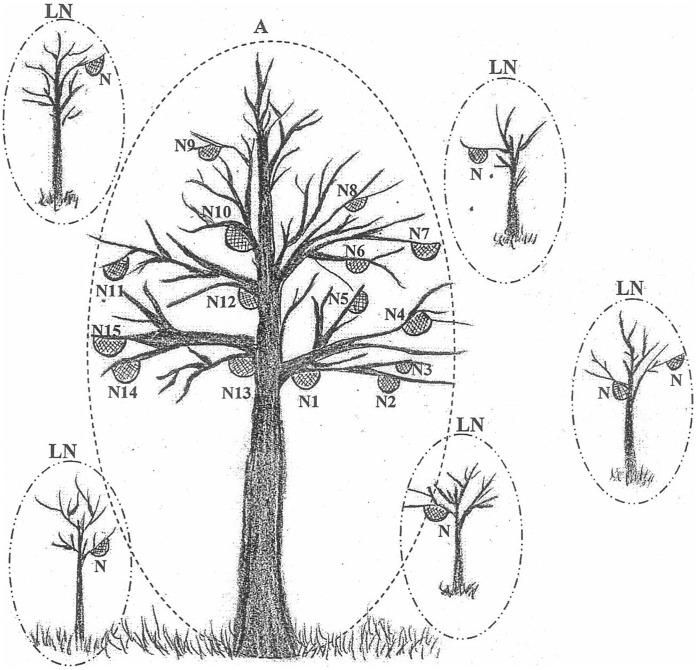
A diagram of *A. dorsata* aggregation (high nesting) and low solitary nesting (Marang, Terengganu, 2010). **A**: An aggregation on a bee tree of ∼ 40 metres in height. **LN**: Low solitary nests on a tree of between 2–5 metres in height. **N**: Nest.

Seasonal migration and aggregation found densely on a bee tree are known as unique characteristics in *A. dorsata*, which may differentiate this particular species from other *Apis* species [4]. The seasonal migration between alternative nesting sites is done to find available forage [5] and control the levels of *Tropilaelaps clarea* (parasitic mite) [6]. While the aggregation structure (up to 50 or 100 nests of *A. dorsata* on a bee tree high in the air [7], usually from 5 to 40 metres above the ground in the rainforest) is a unique feature of this honeybee species, abundance of low aggregations (2 to 10 nests on a tree) and solitary nests (only one or rarely two nests on a tree) are more common in this species [7], especially in the secondary forests in the district of Marang, Terengganu, Malaysia (Figure 1). Both low aggregations and low solitary nests are found on trees of less than 5 metres in height, which makes it easy for honey hunters to harvest these nests. The honey hunters in Marang district climb the tree and remove the whole comb of this type of *A. dorsata* nests easily during the days of harvesting season (Personal observation).

In Malaysia, the interaction between man, the forest and *A. dorsata* has been established for several hundreds of years [8]. Most honey hunters are found in the states of Kedah, Terengganu and Negeri Sembilan in the Peninsular Malaysia [8]. Collection of wild honey is lucrative and generates an income of about RM6,000 (USD2000) per harvesting season in about three months [8]. Because of the inherent organic properties, high medical and nutritional values of wild honey, its price is much higher than the commercial honey, which may be produced by *A. mellifera* and *A. cerana* [9]. Due to the large size of the *A. dorsata* nest, a considerable amount of wild honey (up to 45 kg) [10] may be stored by a nest. This amount of honey tempts local honey hunters to harvest the nests of *A. dorsata* during flowering season frequently. Therefore, Malaysian honey hunters harvest the whole nest of *A. dorsata* by cutting and taking the different parts of a nest for selling. This method of nest harvesting is especially done on solitary nests, which are spread throughout the rainforest of Malaysia. This common harvesting method is deemed unsustainable as the bees may avoid this area as their nesting site in the future causing a decrease of the *A. dorsata* population here.

Honey hunters in Marang claim that they harvest about 600 or more nests of *A. dorsata* per harvesting season (June, July and August) in this area (Personal communication). This figure is seemingly huge, and based on the *A. dorsata* biology and plant flora, it is not possible to have this huge number of *A. dorsata* nests in this area. Furthermore, *A. dorsata* needs specific plant species for providing nectar, pollen and establishment of its nest. Saberioon *et al*. (2010) reported that in Marang, *A. dorsata* constructs its nest on the branches of available nesting support of *Acaia auriculiformis* and *Melaleuca cajuputi* trees which are the sources of nectar and pollen for producing strong flavour and weak density of wild honey in the forested areas of Marang district [11].

We hypothesize that the honey hunters in Marang harvest the same nests, in which the queen of each nest heads away from the original nest site together with her nest members after the first harvest to construct a new nest at another site in the same season and in the available nesting support in the vicinity of the same geographical location. Information on the genetic structure and relatedness among the solitary nests of *A. dorsata* could provide us with the estimation of honey harvesting dates from the new nests to help rectify the current unsustainable nest harvesting practices.

Paar *et al*. (2004) studied the genetic structure and relatedness among and within the *A. dorsata* aggregations in northeast India [5]. Their results revealed significant genetic differentiation among the aggregations. They also found positive relatedness among nests within aggregations which indicated that the colonies within aggregations were more highly related than random colonies. However, they did not study the low solitary nests it was necessary for us to do so since this nest type is mainly harvested in Malaysia.

There is currently no data on the genetic relatedness and population structure of *A. dorsata* solitary nests from Marang district (Terengganu, Malaysia). The knowledge on the genetic structure of *A. dorsata* is needed for effective management and conservation strategies of this species in Malaysia, especially to prevent over harvesting of honey. Overall, the objectives of this study were to estimate the level of intercolonial and intracolonial relatedness and to identify the population structures of Malaysian *A. dorsata* low solitary nests using single locus DNA microsatellites. According to Insuan *et al*. (2007), microsatellites are efficient markers to reveal the detailed genetic structure of *A. dorsata* population [12].

## Results and Discussion

### Microsatellite Amplification and Genetic Variation

All the 15 loci were successfully amplified with the expected sizes of PCR products (Table 1).The inferred genotypes at 15 loci of the queen of each solitary nest are presented in Table 2. The number of the effective alleles displayed per locus (allelic diversity) acts as a useful measure of genetic variability within a population [13]. A total of 134 alleles were found at the 15 microsatellite loci in the honeybees of Marang district population. The number of alleles per locus ranged from 6 alleles for locus Ap273 to 11 alleles for locus Ag005a. All loci were found to be polymorphic. H_o_ ranged from 0.30 to 0.60 across the 15 loci, with mean of 0.41±0.025 and mean of H_e_ 0.82±0.007 (Table 3).

**Table 1:** Detailed information of microsatellite loci.

SSR	Name	Core sequence	Forward primer (5′→3′)	Reverse primer(5′→3′)	Expected size	Reference
**SSR1**	At003	(CT)_6_TT(CT)_14_	GATCATTTCTTTCATTCTTCTCTCTC	ATGCTCGACTATTCCGCG	199	[31]
**SSR2**	A088	(GA)_15_ … (GCTCG)_5_	CGAAGGTAAGGTAAATGGAAC	GGCGGTTAAAGTTCTGG	150	[31]
**SSR3**	AB124	(CT)_8_ (CT)14(GGCT)_8_	GCAACAGGTCGGGTTAGAG	CAGGATAGGGTAGGTAAGCAG	250	[31]
**SSR4**	AB024	(CT)_11_	CACAAGTTCCAACAATGC	CACATTGAGGATGAGCG	100	[31]
**SSR5**	Ag005a	(A)_8_G(A)_6_G(A)_5_	TGTTCCGGCAAGCTGAAG	GTGCTCCGCAACAACGTG	108	[31]
**SSR6**	Ap085	(GA)_6_(GA)_11_	GATCAAACACACAAACGAAAGC	ACCGGAAGCCTAATCAAGG	196	[31]
**SSR7**	Ap226	(CT)_8_	AACGGTGTTCGCGAAACG	AGCCAACTCGTGCGGTCA	231	[31]
**SSR8**	A107	(GCTC)_2_(GCT)_2_(CT)_23_	CCGT GGGAGG58T TTATTGTCG	CCTTCGTAACGGATGACACC	200	[32]
**SSR9< /emph>**	Ap049	(AGG)_7_	CCAATAGCGGCGAGTGTG	GGGCTTCGTACGTCCACC	142	[31]
**SSR10**	Ap243	(TCC)_9_	AATGTCCGCGAGCATCTG	TGTTTACGAGAATTCGACGGG	260	[31]
**SSR11**	Ap273	(CT)_8_	GATCTTGTGTTAAACAGCCG	GATCTCTGGCAGACGAAGAG	108	[31]
**SSR12**	Ad3	(CT)_7_	CCGTAACTGGACTTCTTTCCCTCC	GACAATGGCGTACTTTGTGG	160	[5]
**SSR13**	Ap036	(GA)_21_	CTACGCGCTTACAGGGCA	GCCGAAATTCAACGCTCA	159	[31]
**SSR14**	Ap256	(GA)_12_ AT(GA)_3_	CCAAGTCGCTTCATCATCGT	CCTAAGGTCTACACCCCCGT	162	[31]
**SSR15**	A14	(CT)_13_…(GGT)_9_	GTGTCGCAATCGACGTAACC	GTCGATTACCGATCGTGACG	206	[32]

**Table 2:** Inferred queen genotypes of each solitary nest at each locus (Marang, Terengganu, Malaysia, 2010).

Plot	Queen	SSR 1	SSR 2	SSR 3	SSR 4	SSR 5	SSR 6	SSR 7	SSR 8	SSR 9	SSR 10	SSR 11	SSR 12	SSR 13	SSR 14	SSR 15
A	Nest 1	218/218	150/150	250/250	92/92	110/110	200/200	229/229	204/204	150/150	260/260	110/110	167/167	160/160	206/206	230/230
A	Nest 2	218/220	150/150	250/250	92/92	112/112	200/200	229/229	198/198	142/142	254/260	110/120	157/157	168/168	214/214	214/230
A	Nest 3	218/218	150/150	254/254	100/100	112/112	198/198	233/233	198/198	150/150	254/260	120/120	157/157	160/160	214/214	214/214
A	Nest 4	218/220	150/150	254/254	100/100	112/112	198/198	229/233	204/204	152/152	254/254	120/120	167/167	160/160	214/214	230/230
A	Nest 5	218/218	150/150	254/254	100/100	110/110	200/200	233/233	198/198	150/150	254/260	120/120	157/157	168/168	206/206	230/230
A	Nest 6	218/218	154/154	254/254	100/100	110/110	198/198	233/233	204/204	150/150	260/260	110/110	157/157	160/160	206/214	214/214
A	Nest 7	200/200	154/154	244/244	104/104	100/110	210/210	229/243	206/216	150/160	254/270	114/114	169/175	168/180	200/210	2102/14
A	Nest 8	212/212	148/148	252/252	108/108	118/118	196/196	237/237	206/206	144/144	256/256	116/116	161/169	178/178	216/216	220/220
A	Nest 9	212/212	146/146	254/254	110/110	116/116	196/196	235/237	200/200	148/148	256/262	108/120	169/169	172/178	220/220	220/220
B	Nest 10	212/212	146/146	254/254	108/108	116/116	192/192	235/237	200/200	144/148	256/256	116/116	169/169	172/172	216/216	214/214
B	Nest 11	216/216	148/148	252/252	108/108	118/118	192/196	235/235	206/206	148/148	262/262	108/108	161/161	178/178	216/216	214/214
B	Nest 12	216/216	148/148	254/254	110/110	118/118	196/196	237/237	206/206	148/148	256/256	110/110	161/161	172/172	216/220	220/220
B	Nest 13	212/212	146/146	252/252	110/110	118/118	196/196	235/237	206/206	144/148	262/262	108/108	169/169	172/172	220/220	214/214
B	Nest 14	218/220	140/150	242/246	100/102	104/106	202/204	231/237	212/220	138/146	264/272	112/120	159/171	166/172	202/212	210/216
B	Nest 15	200/210	142/142	246/246	106/106	118/118	210/210	229/243	208/208	138/148	256/260	120/120	173/173	174/180	208/208	224/224
B	Nest 16	212/218	150/156	246/248	102/110	108/114	196/198	231/237	196/206	142/158	254/270	114/120	171/177	168/178	206/218	210/218
B	Nest 17	200/204	148/152	254/256	106/108	104/110	206/210	229/235	216/220	146/160	256/268	108/114	157/169	162/174	202/214	212/226
B	Nest 18	200/204	158/160	244/248	102/104	116/120	196/198	237/245	196/212	140/152	254/264	110/116	167/173	170/170	210/220	216/220
B	Nest 19	218/220	150/154	240/250	92/98	102/110	198/208	233/247	198/218	140/158	254/260	114/120	159/165	166/180	202/216	218/230
C	Nest 20	218/220	150/158	240/250	92/98	102/110	198/208	233/247	198/218	142/158	260/260	114/114	165/167	166/180	202/216	230/230

**Table 3:** A summary of microsatellite genetic variation in the low solitary nests of *A. dorsata*.

Locus	N_a_	N_e_	H_o_	H_e_	PIC
**At003**	7	4.598	0.450	0.783	0.627
**A088**	10	5.096	0.300	0.804	0.656
**AB124**	9	4.819	0.300	0.793	0.640
**AB024**	8	6.504	0.300	0.846	0.621
**Ag005a**	11	5.839	0.350	0.829	0.646
**Ap085**	9	5.369	0.350	0.814	0.672
**Ap226**	8	5.442	0.600	0.816	0.691
**A107**	10	6.667	0.350	0.850	0.709
**Ap049**	10	6.838	0.500	0.854	0.718
**Ap243**	8	5.063	0.600	0.803	0.698
**Ap273**	6	4.469	0.400	0.776	0.648
**Ad3**	10	6.667	0.400	0.850	0.697
**Ap036**	9	6.838	0.400	0.854	0.707
**Ap256**	10	6.557	0.450	0.848	0.666
**A14**	9	5.298	0.400	0.811	0.688
**Mean±SE**	8.933±0.345	5.738±0.223	0.410±0.025	0.822±0.007	0.672±0.0067

N_a_ is the number of different alleles, while N_e_ is the number of effective alleles. H_o_, H_e_ and PIC indicate the observed and expected heterozygosity and polymorphism information content, respectively.

Higher PIC values of a molecular marker indicate higher heterozygote frequency in a population, as well as more genetic information [14]. In this research, all loci showed high polymorphism. It was found that all PIC values were less than their related heterozygosity. The obtained results indicated that the loci with more alleles contained higher rates of heterozygosity and PIC values, but this was not absolute because of the effect of allele frequencies. For example, Ag005a had 11 alleles, while its related heteozygosity and PIC values were lower (H_e_ = 0.829, PIC = 0.646) than locus Ap049 with 10 alleles (H_e_ = 0.854, PIC = 0.718). Thus, it was concluded that at Ap049 locus, the rare alleles contributed little effect on heterozygosity. Furthermore, the heteozygosity at this locus exceeded the expected heterozygosity computed from the number of alleles as a result of mutation drift equilibrium [15]. The mean effective number of alleles (5.738±0.223) across the 15 microsatellite loci indicated that the sample size of this study was enough [16].

A polymorphic locus must have at least 0.10 of heterozygosity to reflect the variation of genetic structure [17]. The mean heterozygosity (0.822±0.007) across the 15 microsatellite loci was sufficient to show genetic variation in the studied population.

### Genetic Structure

The deviations from HWE were assessed using the method of Guo and Thompson (1992) for each locus- nest combination using a Markov chain of 100 000 steps and 1000 dememorization steps [18]. For social insects, the colony structure may show significant deviations from HWE and linkage disequilibrium tests whereas no deviation exists in a population if the workers’ genotypes are used [5]. So, in this research, these analyses were carried out using the queen’s alleles only. The results showed that no nest had significant deviation from HWE (P>0.05). There was also no significant deviation from genotypic linkage disequilibrium (P>0. 34). The results also demonstrated that the low solitary nests which were sampled for this study were mating randomly.

### Population Genetic Differentiation

**Figure 2:**
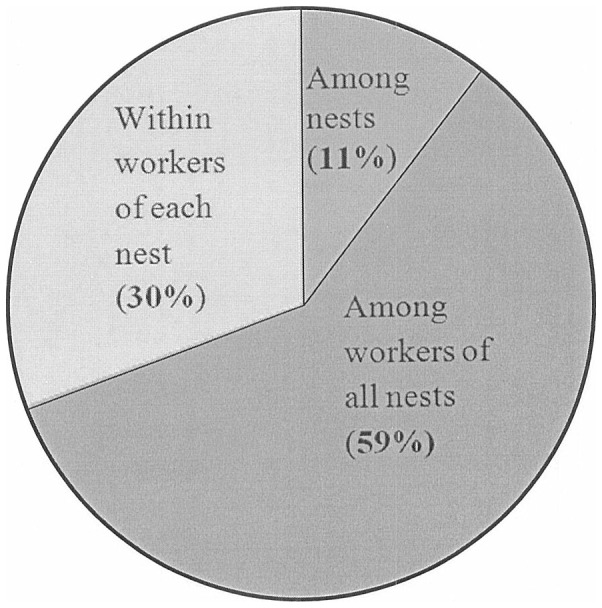
The hierarchical AMOVA based on microsatellite markers for showing the genetic differentiation of *A. dorsata* solitary nests.

Among closely related populations, genetic drift is assumed to be the main factor in genetic differentiation [19]. Hence, F_ST_ index is suggested to be used for estimating the differences between populations [20], [21]. Theoretically, F_ST_ vales change between 0 and 1 [22]. All F_ST_ values between the solitary nests of *A. dorsata* were significantly different from zero (P<0.01). A moderate level of genetic differentiation (F_ST_) among the sampled solitary nests (mean of F_ST_ = 11.6%) was found. In addition, the AMOVA indicated that this percentage of differentiation was due to the differences among the solitary nests (11%), an appreciable amount of genetic variation among workers of all nests (59%), and within workers of each nest (30%), as shown in Figure 2. The low percentage of differentiation (11%) among the solitary nests could support this research hypothesis, which states that the honey hunter may be harvesting the same nest at a different site of a same geographical area and at a different time. The genetic variation among *A. dorsata* workers of all nests and within workers of each nest were resulted as the reproductive system in honeybees and high levels of polyandry [23].

### Genetic Relatedness among the Solitary Nests of *A. dorsata*

We followed Oldroyd *et al*.’s (1995) analytical approach to determine whether the spatial distributions of the solitary nests of *A. dorsata* were random or otherwise [16]. The Poisson method and negative binomial method were used. An organism under Poisson distribution is dispersed randomly, whereas an aggregated organism follows negative binomial distribution in the environment (Dale, 2000 cited in [16]). Both types of distribution were tested, because two types of nests (the solitary and aggregations) were found in the study site. This paper is focused on the results of our work on the more frequently harvested solitary nests. The observed and expected distributions of nests under these models showed that the observed distribution differed significantly (P<0.01) from the negative binomial distribution, indicating that the solitary nests were distributed randomly throughout the plots (Figure 3).

**Figure:**
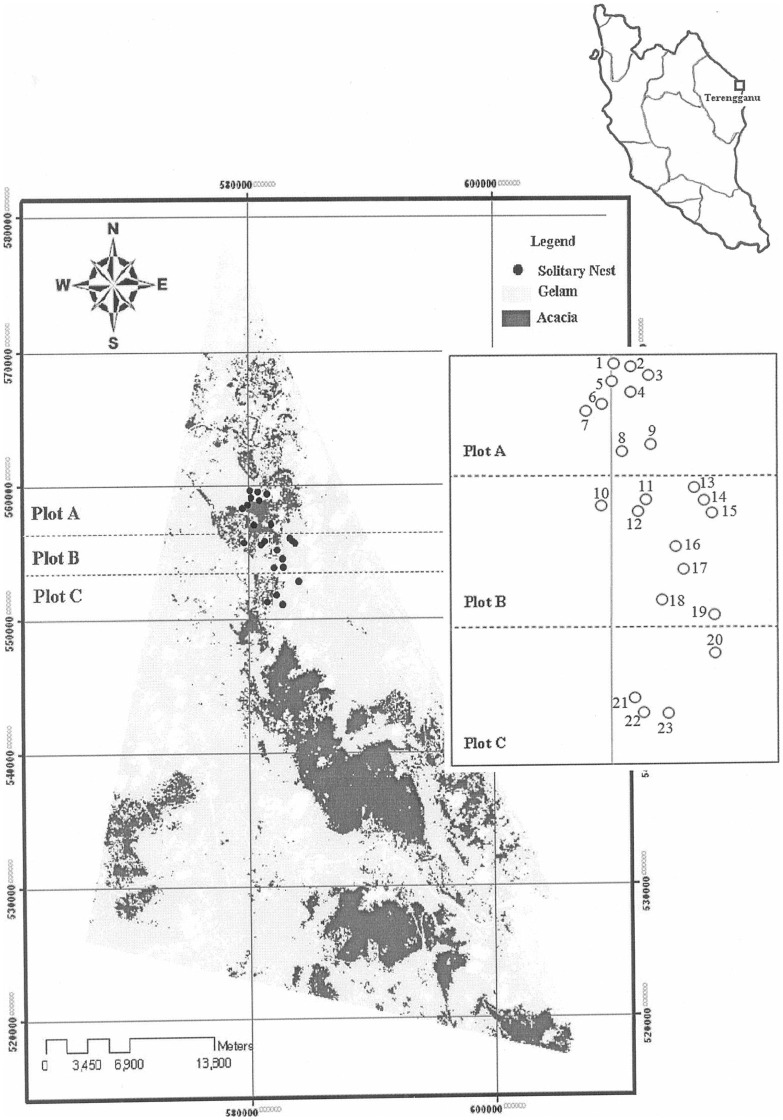
The spatial distribution of *A. dorsata* solitary nests from Marang, Terengganu, Malaysia (2010). Numbers 1 to 20 were the solitary nests, while numbers 21 to 23 were the aggregated nests.

The degree of intercolonial genetic relatedness (R) among the solitary nests of *A. dorsata* revealed positive relatedness between some of the nest pairs based on the queen’s heading of them. The mean relatedness coefficient among the solitary nests of *A. dorsata* was negative (R = −0.053±0.016). The 95% confidence interval was determined by bootstrap resamplings and limits of −0.092 and 0.094 were found. Under Kin selection hypothesis, the majority of relatedness coefficients among pairs of queens should be positive. However, positive values of mean intercolonial relatedness were observed only between 54 pairs of nests out of 190 possible combinations (Table 4). The R values among nest pairs 3–4 and 3–5 was higher than 0.50 showing that their queens were half sibling, whereas nest pair 19–20 showed a relatedness of 0.95 indicating that the same queen was sampled (Table 2 and Table 4).

**Table 4:** Labelled pair wise relatedness matrix [30].

1	2	3	4	5	6	7	8	9	10	11	12	13	14	15	16	17	18	19	20	
0																				**1**
0.36	0																			**2**
0.09	0.34	0																		**3**
0.24	0.22	0.55	0																	**4**
0.36	0.35	0.53	0.28	0																**5**
0.37	0.02	0.58	0.28	0.40	0															**6**
−0.09	−0.10	−0.16	−0.19	−0.09	−0.01	0														**7**
−0.26	−0.28	−0.31	−0.29	−0.30	−0.29	−0.16	0													**8**
−0.28	−0.28	−0.19	−0.16	−0.18	−0.21	−0.20	0.19	0												**9**
−0.26	−0.24	−0.13	−0.20	−0.21	−0.11	−0.13	0.32	0.48	0											**10**
−0.25	−0.22	−0.21	−0.27	−0.29	−0.19	−0.12	0.44	0.06	0.15	0										**11**
−0.19	−0.25	−0.23	−0.21	−0.22	−0.12	−0.20	0.43	0.37	0.13	0.35	0									**12**
−0.27	−0.24	−0.23	−0.29	−0.31	−0.20	−0.09	0.31	0.53	0.28	0.41	0.31	0								**13**
−0.13	−0.11	−0.05	−0.02	−0.04	−0.16	−0.19	−0.19	−0.17	−0.16	−0.24	−0.14	−0.16	0							**14**
−0.13	−0.12	−0.15	−0.13	−0.14	−0.20	0.04	−0.08	−0.11	−0.15	−0.07	−0.04	−0.11	−0.05	0						**15**
−0.13	−0.03	−0.06	−0.04	0.02	−0.18	−0.04	0.02	−0.02	−0.22	−0.14	−0.06	−0.02	0.08	−0.14	0					**16**
−0.15	−0.10	−0.13	−0.14	−0.12	−0.08	0.14	−0.05	−0.06	0.02	−0.01	−0.09	−0.12	−0.10	0.03	−0.30	0				**17**
−0.13	−0.21	−0.21	−0.03	−0.26	−0.15	0.05	0.00	0.02	−0.09	−0.20	0.01	−0.10	−0.04	−0.11	−0.02	−0.19	0			**18**
0.13	0.21	0.10	0.09	0.18	−0.02	−0.13	−0.27	−0.33	−0.28	−0.26	−0.32	−0.35	−0.03	−0.16	0.01	−0.23	−0.21	0		**19**
0.13	0.21	0.10	0.09	0.18	−0.02	−0.13	−0.27	−0.33	−0.28	−0.26	−0.32	−0.35	−0.03	−0.16	0.01	−0.23	−0.21	0.950	0	**20**

The bold numbers (**1–20**) indicate *A. dorsata* solitary nests.

Despite the tendency of *A. dorsata* to aggregate on a single tree which suggested that the colonies within the aggregations might show higher degree of relatedness than random colonies, Paar *et al*. (2004) found that the long distance migration between the original and alternative sites might minimize the genetic differentiation among the geographical locations as well as within the aggregations of this species [5]. Hence, the low genetic differentiation among and within the *A. dorsata* aggregations was due to gene exchange among them. The slightly negative values of intercolony relatedness within the aggregations of *A. dorsata* were found using microsatellites [5]. In their study, no pairs of colonies within the aggregations showed high relatedness. Only one pair of the queens carried at least one identical allele at every locus. These queens were considered to have formed mother and daughter colonies [5]. Paar *et al*. (2004) further explained that the limited number of bee workers per colony and the loci which they used affected their results.

Based on the objectives of the present study, the pair wise nests numbered 19 and 20 showed the highest intercolonial relatedness (R = 0.95) among all the pairs of nests (Table 4) while queen pair carried out two same identical alleles at 10 loci and at least one allele for the remaining five loci. On the contrary, the nest number 16 had the lowest degree of relatedness (R = 0.016) with nest numbers 19 and 20. Despite the distance between plot B and plot C, and the different date of sampling at these sites, 95% of genetic relatedness was found between one pair of nests (numbered 19 and 20). This high level of relatedness between this pair of nests raised the possibility that the honey hunters might have harvested the same nest after the nest shifted to a new site in the same season and at the same geographical area. Hence, it was concluded that the plundering or destruction of a particular *A. dorsata* nest for honey harvesting caused the queen to head the nest members away to establish a new nest at different site but within the same geographical area.

Along the degree of intercolonial relatedness, intracolonial pedigree relatedness (r) was calculated based on the effective number of drones which mated with the queen of each nest (Crozier, 1970 cited in [5]). In the present study, the effective number of drones was found for each nest and locus ranged between 7.89±2.1 and 14.29±3.07, followed by a mean average of intracolonial relatedness of 0.281±0.019. Oldroyd *et al*. (1996) reported that the *A. dorsata* queen mates with an effective number of 20.0±6.6 and a mean average intracolonial relatedness of 0.29±0.007 [23]. They determined the (r) levels using three microsatellites and a large number of workers per nest (42–194). Despite the number of microsatellites and workers per nest used in our study and that of Oldroyd *et al*. (1996), a similar mean average of intracolonial relatedness was found. Our results approach was in line with the view of Takazaki and Nei (1996) [20] that using more microsatellite loci is better for obtaining genetic relationships when the closely related samples are being studied and the average heterozygosity is high.

Our finding on the nesting behaviour of the *A. dorsata* bees sampled along the coastal, submerged *Melaleuca* forest in Marang (Malaysia, Terengganu) should be taken into account by the Forestry Department of Peninsular Malaysia in their effort to formulated possible sustainable methods of nest product harvesting rather than the present practice of cutting off the entire nest.

## Materials and Methods

### Study Area

This study was conducted in Marang district, which is located in the state of Terengganu at the northern east of Peninsular Malaysia, between the upper left of 5°01′ N, 103°11′ E and the lower right of 4°50′ N, 103°24′ E. Mangrove, *Melaleuca*, *Acacia*, rubber and coconut trees are the dominant trees species in this area [11]. The locations of these trees are not in any way restricted information. Hence, no specific permits were required for this study and the authors also confirm that the nests sampled did not involve any endangered or protected species.

In this research, we followed Saberioon *et al*.’s (2010) prediction map for finding the solitary nests of *A. dorsata* in Marang district [11]. The samplings were done during the harvesting season of 2010. The map was prepared for the *A. dorsata* nesting sites [11], and the positions of the solitary nests were added to that map for the present study (Figure 3). The nests numbered 1 to 9, 10 to 19, and 20 to 23 were sampled during the last week of June, third and fourth weeks of July, and third week of August of 2010, respectively. The distribution data of the solitary nests is shown in and Table 5. The nests numbered 1 to 20 were solitary nests, while those numbered 21 to 23 were the aggregated nests on three different bee trees (i.e. up to 20 nests on a bee tree). Therefore, in this study, only the low solitary nests were analyzed to achieve the research objectives and kept the aggregated nests for the next research. Furthermore, one-kilometre distance was estimated as the forage area of each nest [11]. Then, at least one-kilometre distance was kept between the pair wise nests of each plot (Figure 3).

**Table 5:** The distribution data of low solitary nests of *Apis dorsata* in Marang district.

Nest	Coordinate X	Coordinate Y	Number of nest on tree
1	584212.23	551979.28	1
2	585361.07	551090.93	1
3	584449.37	551973.10	1
4	583015.41	556065.82	1
5	578453.71	558196.83	1
6	581476.68	560048.02	1
7	584303.09	555828.73	1
8	581075.12	557498.23	1
9	590707.35	544311.77	1
10	581651.14	557439.59	1
11	581108.79	556896.12	1
12	583409.25	554716.91	1
13	583095.50	556114.89	1
14	593358.41	538489.08	1
15	583132.63	556317.61	1
16	593108.59	537899.25	1
17	583905.88	556283.56	1
18	576381.47	560296.06	1
19	583083.15	556010.49	1
20	582379.28	552542.33	1

### Sampling and DNA Extraction

Based on the effective number of mating in *A. dorsata* (20.0±6.6) [23], 20 bee workers per nest were collected. The entire nests were removed and the bee workers were taken from the honey part of each nest separately. The bee workers were preserved at 95% of ethanol and stored at −80°C for DNA extraction later.

The total genomic DNA was isolated from the thorax of each bee worker of each nest using the Wizard® Genomic DNA Purification Kit (Promega, USA). The protocol of DNA extraction followed the animal tissue protocol, which is available at http://www. promega.com/resources/protocols/. The concentrations of the extracted DNA stocks were measured using a spectrophotometer (Eppendorf Biophotometer Plus). The concentrations of DNA ranged from 200–370 ng. Then, the needed volume of DNA stocks was diluted to obtain 10 ng/ul concentration of working DNA. After testing for the purity and dilution, all the DNAs were labelled. So that each DNA sample can be traced to the worker and the nest from this was originated. The DNAs were kept at −20°C for PCR amplification later.

### Analysis of Microsatellite Polymorphisms

Twenty two (22) heterospecific primer sets of DNA microsatellites (including Ad3, A007, AB024, A088, AB124, Ap036, Ap043, Ap049, Ap068, Ap085, Ap207, Ap226, Ap243, Ap273, Ap297, At003, Ag005a, A107, A76, B124 and A14) were screened and 15 polymorphic loci (Table 1) were selected for achieving the objectives of the research using the reaction conditions described in Paar *et al*. (2004). In the present research, however, the non-labelled primers were used. The loci were size fractionated on 8% of denaturating polyacrylamide gels, visualized by a modified silver staining protocol [24] and then their sizes were estimated by a comparison to a 20-bp ladder marker (Fermentas). The fragments were scored using the UVIDoc version 99.02 software. The DNA fragment sizes as the base pairs were entered into the Excel sheets as co-dominant markers and were saved for data analysis.

### Statistical Analysis

In social insects that follow the haplodiploidy sex determination system, the genetic structure may be determined using the genotypes of the queen and paternal genotypes. In this study, the workers’ genotypes per nest of *A. dorsata* were converted to COLONY [25] software format. This software determines the inferred genotypes of the queen and paternal genotypes of each nest. The inferred genotypes were subjected to genetic analysis. The inferred queen’s genotype of each nest was conducted to calculate the number of alleles per locus (N_a_), effective number of alleles (N_e_), observed and expected heterozygosity (H_o_ and H_e_), polymorphism information content (PIC), and relatedness (R) among the queens which head the low solitary nests separately. The genetic differentiation measure (F_ST_) was estimated using the paternal inferred genotypes. According to Paar *et al*. (2004), the paternal genotypes were diploidized as co-dominant marker. GENALEX version 6.41 [26] was used to estimate the following measures of genetic variation N_a_, N_e_, H_o_, H_e_, and R, and genetic differentiation structure (F_ST_). Hierarchical genetic structuring based on the analysis of molecular variance (AMOVA) [27] and bootstrap resampling were executed by using GENALEX version 6.41 [26]. Meanwhile, PIC values were calculated using the microsatellite toolkit package [28]. Hardy Weinberg equilibrium (HWE) tests and genotypic linkage disequilibrium were performed using GENEPOP version 3.3 [29], whereas the intercolonial relatedness between the solitary nests of *A. dorsata* was computed based on the queen’s inferred genotype using an algorithm as described by Queller and Goodnight (1989) [30].

### Conclusion

This study was conducted to show the genetic relatedness among the solitary nests of *A. dorsata* in the Marang district, Malaysia. The results revealed the high intracolonial relatedness within the studied nests based on the data obtained from the use of 15 single locus DNA microsatellite markers. The presence of appreciable levels of intercolonial relatedness between several single pair of nests indicated the necessity for a comprehensive study involving a large sample size to confirm whether the honey hunters in this area of Malaysia harvest the same nest at a different sites and dates during a single harvesting season or not. Such research is needed to formulate effective management strategies to replace the current common unsustainable *A. dorsata* nest harvesting practice of whole solitary nest removal. Genetic studies on the aggregate nests should also be done in the future.

## References

[1] Oldroyd BP, Wongsiri S (2006). Asian honey bees (biology, conservation, and human interactions).. Harvard University Press 340 p.

[2] Mardan M (1989). Thermoregulation in the Asiatic giant honeybee *Apis dorsata* (Hymenoptera: Apidae).. Ph.D. dissertation, University of Guelph, Ontario.

[3] Mardan M (2007). More about Malaysia bees and bee trees.. Bees for Development.

[4] Kastberger G, Sharma DK (2000). The predator-prey interaction between blue- bearded bee eaters (Nyctyornis athertoni Jardine and Selby 1830) and giant honeybees (*Apis dorsata* Fabricius 1798).. Apidologie.

[5] Paar J, Oldroyd BP, Huettinger E, Kastberger G (2004). Genetic structure of an *Apis dorsata* population: the significance of migration and colony aggregation.. Journal of Heredity.

[6] Rinderer T, Oldroyd B, Lekprayoon C, Wongsiri S, Boonthai C (1994). Extended survival of the parasitic honey bee mite *Tropilaelaps clareae* on adult workers of *Apis mellifera* and *Apis dorsata*.. Journal of Apicultural Research.

[7] Oldroyd B, Osborne K, Mardan M (2000). Colony relatedness in aggregations of *Apis dorsata* Fabricius (Hymenoptera, Apidae).. Insectes Sociaux.

[8] Hamid AH (1997). Honey or money: Changing nature of honey collectors- environment relationship in rural Malaysia.. In: Proceedings of the International Conference on Tropical Bees: The environment Universiti Pertanian Malaysia (Mardan M, Sipat A, Yusoff KM, Kiew HMS, Abdullah MM, eds).

[9] Yong PL, Haji Othman MS (2007). Economic values of honey bees, Peninsular Malaysia. Forestry Department Peninsular Malaysia (FDPM). Mashka sdn.. Bhd.

[10] Ruttner F (1988). Biogeography and taxonomy of honeybees.. Berlin: Springer-Verlag.

[11] Saberioon MM, Mardan M, Nordin L, Alias MS, Gholizadeh A (2010). Predict location(s) of *Apis dorsata* nesting sites using remote sensing and geographic information system in Melaleuca forest.. American Journal of Applied Sciences 7.

[12] Insuan S, Deowanish S, Klinbunga S, Sittipraneed S, Sylvester HA (2007). Genetic differentiation of the giant honey bee (*Apis dorsata*) in Thailand analyzed by mitochondrial genes and microsatellites.. Biochemical Genetics.

[13] Norris AT, Bradley DG, Cunningham EP (1999). Microsatellite genetic variation between and within farmed and wild Atlantic salmon (*Salmo salar*) populations.. Aquaculture.

[14] Ting J, Ling Y, Wen-bin B, Guo-hong C (2008). Study on genetic diversity of *Apis cerana cerana* and *Apis mellifera ligustica* in China with microsatellite markers. Research Journal of.. Animal Sciences.

[15] Cornuet JM, Luikart G (1997). Description and power analysis of two tests for detecting recent population bottlenecks from allele frequency data.. Genetics.

[16] Oldroyd BP, Smolenski A, Lawler S, Estoup A, Crozier R (1995). Colony aggregations in *Apis mellifera*.. L. Apidologie.

[17] Ott J (2001). Analysis of human genetic linkage (revised edition).. Baltimore: Johns Hopkins University Press.

[18] Guo SW, Thompson EA (1992). Performing the exact test of Hardy - Weinberg proportion for multiple alleles.. Biometrics.

[19] Weir BS, Cockerham CC (1984). Estimating F-Statistics for the analysis of population structure.. Evolution.

[20] Takezaki N, Nei M (1996). Genetic distances and reconstruction of phylogenetic trees from microsatellite DNA.. Genetics.

[21] Weir BS (1990). Genetic data analysis.. Sinauer Associates, Sunderland, USA. pp 222–289.

[22] Hartl DL, Clark AG (2007). Principles of population genetics. Sinauer Associates Inc. Publishers. Sunderland, Massachussettes.. 652 p.

[23] Oldroyd BP, Smolenski AJ, Cornuet J, Wongsiri S, Estoup A (1996). Levels of polyandry and intracolonial genetic relationships in *Apis dorsata* (Hymenoptera: Apidae).. Annals of the Entomological Society of America.

[24] Creste S, Tulmann NA, Figueira A (2001). Detection of single sequence repeat polymorphisms in denaturing polyacrylamide sequencing gels by silver staining.. Plant Molecular Biology Reporter.

[25] Wang J (2008). COLONY. Version 2.0.1.1. Zoological Society of London website.. http://www.zsl.org/science/research-projects/software/colony,1154,AR.html.

[26] Peakall R, Smouse PE (2010). GenAlEx 6.41: Genetic analysis in Excel. Population genetic software for teaching and research. The Australian National University website (Canberra, Australia).. http://www.anu.edu.au/BoZo/GenAlEx/.

[27] Michalakis Y, Excoffier L (1996). A generic estimation of population subdivision using distances between alleles with special reference for microsatellite loci.. Genetics.

[28] Park S (2008). Excel Microsatellite Toolkit. Version 3.1.1. Animal Genomics Lab website, (University College, Dublin, Ireland).. http://animalgenomics.ucd.ie/sdepark/ms-toolkit/.

[29] Raymond M, Rousset F (1995). GENEPOP (version 3.3): population genetics software for exact tests and ecumenicism.. Journal of Heredity.

[30] Queller D, Goodnight KF (1989). Estimating relatedness using genetic markers.. Evolution.

[31] Solignac M, Vautrin D, Loseau A, Mougel F, Baudry E (2003). Five hundred and fifty microsatellite markers for the study of the honeybee (*Apis mellifera* L.) genome.. Molecular Ecology Notes.

[32] Estoup A, Garnery L, Solignac M, Cornuet JM (1995). Microsatellite variation in honeybee (*Apis mellifera* L.) populations: hierarchical genetic structure and test of the infinite allele and stepwise mutation models.. Genetics.

